# Stimulation of the tibial nerve—a randomised trial for urinary problems associated with Parkinson’s—the STARTUP trial

**DOI:** 10.1093/ageing/afac114

**Published:** 2022-06-15

**Authors:** Doreen McClurg, Andrew Elders, Suzanne Hagen, Helen Mason, Jo Booth, Anne-Louise Cunnington, Richard Walker, Katherine Deane, Danielle Harari, Jalesh Panicker, Susan Stratton, Jaclyn McArthur, Ceri Sellers, Marissa Collins

**Affiliations:** NMAHP RU, Glasgow Caledonian University, Glasgow G4 0BA, UK; NMAHP RU, Glasgow Caledonian University, Glasgow G4 0BA, UK; NMAHP RU, Glasgow Caledonian University, Glasgow G4 0BA, UK; Yunus Centre for Social Business and Health, Glasgow Caledonian University, Glasgow G4 0BA, UK; Scholl of Health & Life Sciences, Glasgow Caledonian University, Glasgow G4 0BA, UK; The New Stobhill Hospital – NHSGGC, G21 3UW; Northumbria Healthcare NHS Foundation Trust, NE25 0QJ; School of Nursing Sciences, University of East Anglia, NR4 7TJ, Norwich; Guy’s & St Thomas Hospital NHS Foundation Trust, SE1 7EH, London; University College London Hospitals NHS Foundation Trust, WC1E 6BT, London; NMAHP RU, Glasgow Caledonian University, Glasgow G4 0BA, UK; Yunus Centre for Social Business and Health, Glasgow Caledonian University, Glasgow G4 0BA, UK; NMAHP RU, Glasgow Caledonian University, Glasgow G4 0BA, UK; Yunus Centre for Social Business and Health, Glasgow Caledonian University, Glasgow G4 0BA, UK

**Keywords:** neurology, bladder dysfunction, neuromodulation, Parkinson’s, older people

## Abstract

**Background:**

non-motor symptoms such as bladder dysfunction are common (80%) in people with Parkinson’s increasing the risk for falls with a negative impact on health-related costs and quality of life.

We undertook STARTUP to evaluate the clinical and cost-effectiveness of using an adhesive electrode to stimulate the transcutaneous tibial nerve stimulation (TTNS) to treat bladder dysfunction in people with Parkinson’s disease (PD).

Study design, materials and methods: STARTUP was a parallel two-arm, multi-centre, pragmatic, double-blind, randomised controlled trial. Each participant attended one clinic visit to complete consent, be randomised using a computer-generated system and to be shown how to use the device.

The trial had two co-primary outcome measures: International Consultation on Incontinence Questionnaire-Urinary Incontinence Short Form and the International Prostate Symptom Score (IPSS). These were completed at baseline, 6 and 12 weeks. A bladder frequency chart and resource questionnaire were also completed.

**Results:**

two hundred forty two participants were randomised. About 59% of participants were male, the mean age was 69 years and mean time since diagnosis was 6 years. Questionnaire return rate was between 79 and 90%.

There was a statistically significantly lower score in the active group at 6 weeks in the IPSS questionnaire (mean difference (Standard deviation, SD) 12.5 (6.5) vs 10.9 (5.5), effect size −1.49, 95% CI −2.72, −0.25). There was no statistically significant change in any other outcome.

**Conclusion:**

TTNS was demonstrated to be safe with a high level of compliance. There was a significant change in one of the co-primary outcome measures at the end of the treatment period (i.e. 6 weeks), which could indicate a benefit. Further fully powered RCTs are required to determine effective treatments.

## Key Points

- Bladder symptoms are common in people who have Parkinson’s.
- There are few evidence based treatments.
- Transcutaneous tibial nerve stimulation demonstrated only a very limited effect on bladder symptoms.
- There were no adverse side effects and adherence was high.

## Background

Parkinson’s disease (PD) is characterised predominantly by motor complaints, such as slow and restricted walking, rigidity and tremor. The reported prevalence of lower urinary tract symptoms (LUTS) in people with Parkinson’s (PwP) ranges from 38 to 71% and is largely manifested by storage/overactive bladder (OAB) symptoms, which include nocturia (>60%), urinary urgency (33–54%), daytime frequency (16–35%) and urinary incontinence (26–28%; [1]). The latter may be in part functional if immobility, cognitive impairment or poor manual dexterity complicates the situation. LUT symptoms have been shown to exacerbate the risk for falls, fractures and decreased quality of life [2].

Current treatments for LUTS in PwP are limited, with very few high quality studies on effectiveness. It is likely that levodopa and other Parkinson’s medication affect bladder function; however, studies evaluating the effects of these medications on micturition have produced conflicting results [1]. Currently, PwP may be offered advice on fluid intake and behavioural treatment such as habit training or timed voiding with or without pelvic floor muscle training, but the evidence base supporting these techniques is limited. Anticholinergics are generally used as a first-line treatment for OAB symptoms. However, it is important to balance the therapeutic benefits of these drugs with the reported adverse effects, such as dry mouth, constipation, increased risk of higher post-void residuals and their potential effect on cognitive function, and caution in prescribing is advised [3, 4]. Intravesical injection of botulinum toxin into the detrusor is sometimes considered in PwP and has been described in four small studies to date, which reported some improvement in symptoms [5–8]. For the reasons discussed, there is a need to explore options that are non-invasive and associated with minimal side effects.

The tibial nerve is a mixed nerve containing sensory and motor fibres of L4–S3, and it originates from the same segments of the spinal cord as the innervation to the bladder, pelvic floor and rectum. There is some evidence that stimulating this nerve with low-frequency pulses at a tolerable intensity can potentially treat OAB-related symptoms [9–11]. Although the exact mechanism is not yet fully understood [12–15], this stimulation is thought to increase inhibition of the spinothalamic neurons within the spinal cord, reducing firing input to the pontine micturition centres (PMC) and inhibiting the information facilitated by those nerves to the bladder [16–20]. Percutaneous stimulation (using a small gauge needle) or transcutaneous stimulation (using a sticky electrode just behind the medial malleolus) are the primary methods of delivering the stimulation produced by a small hand held unit.

## Objectives

The STARTUP trial was a fully powered two groups (Intervention vs Placebo) double-blind RCT to assess if transcutaneous tibial nerve stimulation (TTNS) was an effective therapeutic option for the treatment of OAB symptoms in PwP.

## Methods

A published protocol is available for the trial [21]. Participants were recruited from 12 UK community outpatient care settings where PD care was usually provided. Additional help with recruitment was provided by Parkinson’s UK, who promoted the trial on their website. Clinicians delivering the intervention at the sites were trained by the STARTUP trial team in the trial processes and intervention delivery.

Following the provision of study information participants attended for one trial visit where inclusion/exclusion criteria were checked, and questions were answered prior to them giving informed consent.

### Inclusion criteria

- Patients of >18 years of age with a diagnosis of Parkinson’s and self-reported problematic LUT symptoms.
- Capacity to consent and comply with the protocol.
- Stable Parkinson’s medication for 3 months.
- Participants who were treatment naïve, failed or continuing treatment with antimuscarinic medication (group allocation was minimised to account for these groups).
- Patients who were being or had been treated for benign prostatic hyperplasia or prostate cancer could be included at the discretion of the PI.
- Patients taking medications such as alpha-blocker medicines could be included at the discretion of the PI.
- Patients who were being or had been treated for cancer (e.g. urological cancer) could be included on an individual basis at the discretion of the PI.

### Exclusion criteria

- Pacemaker or implanted electrical device, including deep brain stimulation.
- Ulceration or broken skin in area of pad placement.
- History of peripheral vascular disease.
- History of epilepsy.
- Current urinary tract infection.
- Receipt of Botox for bladder symptoms or TTNS within the last year.
- Unable to understand the instructions relating to the bladder diary and/or the use of the stimulator or does not have a relative willing to help.

### Randomisation

Group allocation was carried out remotely via a web-based automated application provided by the clinical trials unit in Aberdeen. The allocation ratio was 1:1 with minimisation by (i) severity of urinary symptoms, using the International Prostate Symptom Score (IPSS; [22, 23]), at baseline and (ii) the status on antimuscarinic medication, i.e. treatment naïve, failed or continuing such treatment. Participants and those who undertook data input and analysis were blind to group allocation.

### Sample size

Please see protocol for the full justification of our sample size [21]. Monitoring of accruing data indicated better retention rates but greater variance in the outcome measures than had been originally assumed. The original sample size of 208 [21] was revised to 236 to allow for retention of 85% and standard deviation (SDs) of 5.5 and 6.5 for International Consultation on Incontinence Questionnaire-Urinary Incontinence Short Form (ICIQ-UI SF) and IPSS respectively. This amendment was approved in November 2019 (GN18RE170 REC reference: 18/ES/00420 and by our Trial Steering Committee).

### Outcome measures

Participants completed questionnaires at baseline (pre-treatment), 6 and 12 weeks.

The trial had two co-primary outcome measures: ICIQ-UI SF [24] and the IPSS [22, 23], which are validated for use in males and females.

The ICIQ-UI SF assesses the impact of symptoms of incontinence on quality of life and outcome of treatment. It consists of four questions measuring frequency of urinary incontinence (UI), amount of leakage, overall impact of UI and when leakage occurs. The total score ranges from 0 to 21 with higher values indicating increased severity of symptoms [24].

The IPSS is based on the answers to seven questions concerning OAB symptoms. Questions 1–7 refer to: incomplete emptying; frequency; intermittency; urgency; weak stream; straining and nocturia. Question eight refers to the patient’s perceived quality of life. Each question is assigned points from 0 to 5 indicating increasing severity of the particular symptom. The total score ranges from 0 to 35 (asymptomatic to very symptomatic). Patients can be tentatively classified: 0–7 = mildly symptomatic; 8–19 = moderately symptomatic and 20–35 = severely symptomatic [22, 23].

Secondary outcome measures included the SF-Qualiveen questionnaire [25], and the Parkinson’s Disease Quality of Life Questionnaire (PDQ-8) [26].

Participants also completed a 72-h Bladder diary recording micturition frequency, leakage episodes and urgency.

Resource use relating to bladder symptoms was also monitored at the 6 and 12 week time-points, with questions relating to visits to the doctor/nurse/hospital, medications bought/prescribed and purchases such as continence pads. An economic evaluation considered the cost-effectiveness of providing TTNS for PwP and OAB symptoms compared with placebo from a health sector perspective.

Participant experience and protocol fidelity was assessed at 6 weeks in a brief quantitative exit questionnaire. All diaries and questionnaires were returned to the trial office in a reply-paid envelope.

### Intervention

Following randomisation participants were shown how to use the device according to group allocation. The treatment protocol developed by Amarenco *et al*. [27] was followed. Participants allocated to the intervention group were asked to place the self-adhesive surface electrode on either leg, 2-cm behind the medial malleolus (inside of ankle) and the positive electrode 10-cm proximal to it. The stimulation frequency was delivered at a fixed frequency of 10 Hz and pulse width of 200 μs in continuous mode. Setting of the intensity level of stimulation current (range 0–50 mA) was guided by the motor (flexion of the big toe) or sensory response (paraesthesias over the sole).

Participants randomised to the placebo group were asked to place the electrodes on the lateral side of the leg (outside of ankle) thus avoiding the tibial nerve and relevant cutaneous nerves. The stimulation current was pre-set at 2 Hz, and participants were advised to turn the unit up until they felt a slight sensation and then turn it down to 2 mA.

The clinician set the parameters and locked the TTNS unit to ensure participants were unable to change the settings or delete data. All equipment and an instruction booklet were issued to the participant to take home. All participants were advised to use the stimulator for two 30-min sessions per week for 6 weeks. The stimulation unit recorded how often and for how long the participant used it during the 6 weeks of intervention. This usage information was downloaded when the unit was returned to centre staff.

Participants were phoned weekly to ascertain fidelity. A final follow-up call was made at week 12 to ascertain the success or otherwise of blinding.

## Results

**Figure 1:**
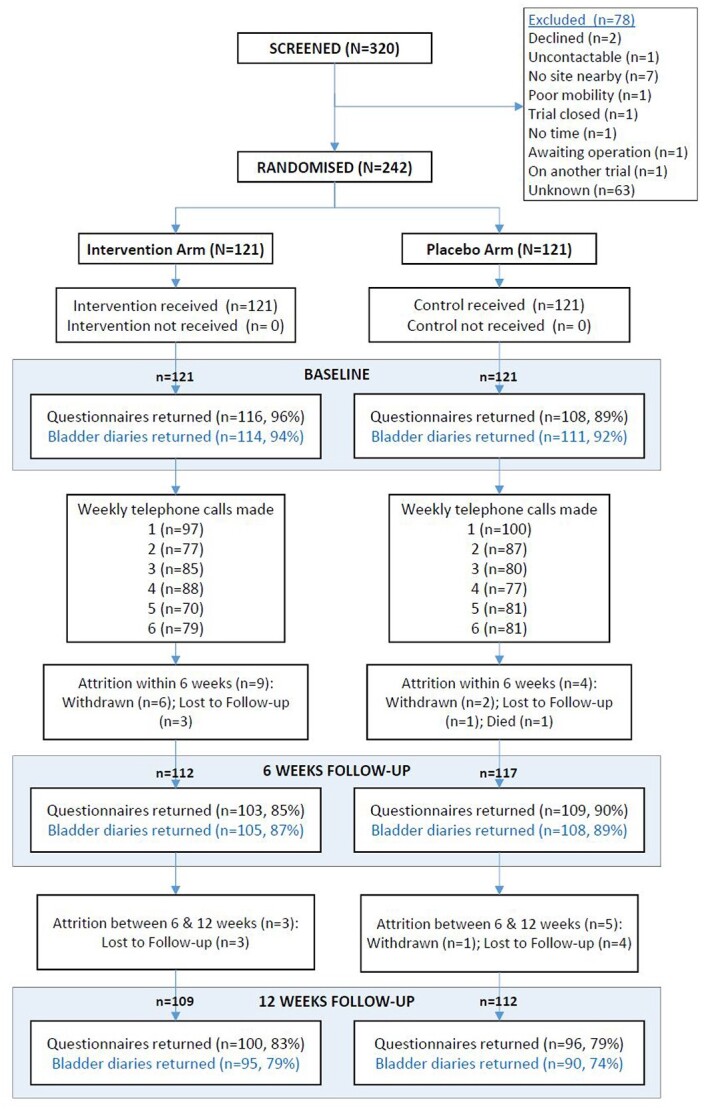
Consort diagram.

A total of 242 participants were randomised, 121 in the intervention group and 121 to receive placebo TTNS. The mean age was 69 years (SD 8.6), 59% (*n* = 121) of participants were male and median time from diagnosis was 5 years (interquartile range, IQR 6.3). The two randomised groups were comparable at baseline (Table 1 and Figure 1).

**Table 1:** Baseline characteristics

		**Placebo arm**			**Intervention arm**		
**Demographics**							
Age (years)	*N*, Mean, SD	119	69.5	8.7	117	68.7	8.5
**Male**	*N, n*, %	121	69	57.0%	121	74	61.2%
**Parkinson’s characteristics**							
Time since Parkinson’s diagnosis (years)	*N*, Med, IQR	119	5.1	6.5	118	5.1	6.1
Antimuscarinic medication status							
Continuing	*N, n*, %	121	37	30.6%	121	38	31.4%
Failed	*N, n*, %	121	16	13.2%	121	17	14.1%
Naïve	*N, n*, %	121	68	56.2%	121	66	54.5%
**Bladder symptoms**							
Duration of bladder symptoms (years)	*N*, Med, IQR	119	2.9	4.8	118	3.5	4.7
Severity of bladder symptoms (IPSS)							
None	*N, n*, %	121	0	0%	121	0	0%
Mild	*N, n*, %	121	17	14.0%	121	16	13.2%
Moderate	*N, n*, %	121	82	67.8%	121	85	70.3%
Severe	*N, n*, %	121	22	18.2%	121	20	16.5%
Incomplete emptying (any)	*N, n*, %	121	72	59.5%	121	86	71.1%
Urine leakage (any)	*N, n*, %	108	92	85.2%	116	102	87.9%
Urinary urgency (any)	*N, n*, %	121	115	95.0%	121	112	92.6%
**Bowel symptoms**							
Severity of bowel symptoms (any)	*N, n*, %	119	85	71.4%	118	74	62.7%
Constipation (any)	*N, n*, %	120	69	57.5%	120	59	49.2%
Bowel strain (any)	*N, n*, %	88	55	62.5%	81	57	70.4%
Wind (any)	*N, n*, %	89	42	47.2%	81	36	44.4%
Leakage of stool (any)	*N, n*, %	86	12	14.0%	77	15	19.5%

Follow-up questionnaire return rates were 93% at baseline, 88% at 6 weeks and 81% at 12 weeks. Attrition was low and similar in both groups. At 6 weeks, eight participants had withdrawn, four were lost to follow-up and one had died. At 12 weeks there was one further withdrawal and seven lost to follow-up.

The statistical analysis used an intention to treat approach. The primary outcome analysis indicated there was no significant difference between the two groups in the severity of ICIQ-UI SF score at any time-point (Table 2). Analysis of the co-primary outcome measure indicated that IPSS was statistically significantly lower in the active TTNS group at 6 weeks (mean difference (SD) 12.5 (6.5) vs 10.9 (5.5), effect size −1.49, 95% CI −2.72, −0.25). A difference of 3 points is thought to be clinically meaningful [23], hence this was not clinically significant. There was no statistically significant difference at 12 weeks.

**Table 2:** Primary outcomes

		**Baseline**		**6 weeks**			**12 weeks**		
		**Placebo arm**	**Intervention arm**	**Placebo arm**	**Intervention arm**	**Effect size** MD (95% CI)	**Placebo arm**	**Intervention arm**	**Effect size** MD (95% CI)
**ICIQ-UI SF** Score*	Mean (SD)	***N* = 108** 8.8 (5.3)	***N* = 115** 8.6 (4.9)	***N* = 109** 8.4 (5.0)	***N* = 101** 7.9 (5.0)	−0.29 (−1.13, 0.55) *P* = 0.495	***N* = 94** 8.7 (5.1)	***N* = 99** 8.1 (5.1)	−0.55 (−1.47, 0. 37) *P* = 0.241
**Symptom severity** (based on ICIQ-UI SF)									
None (0) Slight (1–5) Moderate (6–12) Severe (13–18) Very severe (19–21)	*n*, % *n*, % *n*, % *n*, % *n*, %	14, 13.0% 14, 13.0% 53, 49.1% 26, 24.1% 1, 0.9%	13, 11.3% 15, 13.0% 65, 56.5% 20, 17.4% 2, 1.7%	13, 11.9% 20, 18.4% 47, 43.1% 28, 25.7% 1, 0.9%	11, 10.9% 21, 20.8% 50, 49.5% 17, 16.8% 2, 2.0%		8, 8.5% 20, 21.3% 40, 42.6% 25, 26.6% 1, 1.1%	11, 11.1% 23, 23.2% 41, 41.4% 22, 22.2% 2, 2.0%	
**I-PSS** Score*	Mean (SD)	***N* = 121** 13.6 (5.9)	***N* = 121** 13.4 (5.3)	***N* = 106** 12.5 (6.5)	***N* = 97** 10.9 (5.5)	−1.49 (−2.72, −0.25) *P* = 0.018	***N* = 89** 11.6 (6.0)	***N* = 95** 11.6 (5.8)	−0.09 (−1.37, 1.19) *P* = 0.885
**Symptom severity** (based on I-PSS)									
Mild (0–7) Moderate (8–19) Severe (20–35)	*n*, % *n*, % *n*, %	17, 14.1% 82, 67.8% 22, 18.2%	16, 13.2% 85, 70.3% 20, 16.5%	30, 28.3% 58, 54.7% 18, 17.0%	32, 33.0% 57, 58.8% 8, 8.3%		24, 27.0% 55, 61.8% 10, 11.2%	27, 28.4% 60, 63.2% 8, 8.4%	
**I-PSS quality of life** **Score**	Mean (SD)	***N* = 121** 4.1 (1.3)	***N* = 121** 4.0 (1.2)	***N* = 108** 3.5 (1.4)	***N* = 103** 3.6 (1.4)		***N* = 92** 3.4 (1.5)	***N* = 98** 3.4 (1.5)	
Delighted Pleased Mostly satisfied Mixed Mostly dissatisfied Unhappy Terrible	*n*, % *n*, % *n*, % *n*, % *n*, % *n*, % *n*, %	1, 0.8% 2, 1.7% 12, 9.9% 27, 22.3% 25, 20.7% 40, 33.1% 14, 11.6%	0, 0.0% 1, 0.8% 13, 10.7% 30, 24.8% 32, 26.5% 35, 28.9% 10, 8.3%	2, 1.9% 5, 4.6% 19, 17.6% 33, 30.6% 21, 19.4% 17, 15.7% 11, 10.2%	1, 1.0% 4, 3.9% 19, 18.5% 28, 27.2% 23, 22.3% 20, 19.4% 8, 7.8%		2, 2.2% 4, 4.4% 20, 21.7% 26, 28.3% 13, 14.1% 20, 21.7% 7, 7.6%	3, 3.1% 7, 7.1% 15, 15.3% 32, 32.7% 15, 15.3% 18, 18.4% 8, 8.2%	

Analysis of secondary outcomes (Appendices 1 and 2, Supplementary data are available in *Age and Ageing* online) indicated there were no significant differences between groups in frequency of micturition (bladder diary) or quality of life measures (SF-Qualiveen, PDQ-8). The number of daily micturition episodes were ~9 per day during all three phases. There was a small but not significant difference in the number of daily urgency episodes and the number of daily leakage episodes in favour of the active TTNS group.

The participant experience of using the TTNS device was positive with 90% saying it was easy to use and 80% saying they were willing to continue using the device. Recorded use of the stimulator reported an average use of 5.7 (SD 1.5) hours in each group per week (recommended 6 h per week) with complete usage of unit i.e. 6 h in over 65% of participants in each group. About 52% of the intervention group reported that they thought they were in the intervention group and 73% of the control group thought they were in the control group. There were no related adverse events.

### Subgroup analysis/sensitivity analysis

Post-hoc subgroup analyses were undertaken for the two primary outcomes using: age (<65 and +65); gender; antimuscarinic drug use status (naïve, continuing and failed) and symptom severity.

There were no significant subgroup interactions. The age subgroup analysis showed some difference in IPSS at 6 weeks, with those ≥65 years showing greater improvement than <65 years, but this was not significant at the stricter level of 1% required for this analysis.

### Cost-effectiveness analysis

Resource use questionnaires were used to calculate average NHS resource use and data on the cost of the intervention and placebo intervention were collected. Table 3 details the number of appointments attended by those in each group and the unit cost and source used in the cost-effectiveness analysis.

**Table 3:** Resource use for bladder symptoms with unit costs

**Type of appointment**	**Placebo**	**Intervention**	**Unit cost**	**Source for unit cost**
Accident and emergency	6 (0.03)	1 (0 0.005)	£133	ISD Scotland, April 2018 to March 2019, A&E cost per attendance, average of all boards; https://www.isdscotland.org/health-topics/finance/costs/Detailed-Tables/index.asp
Hospital overnight stay	0	0	£1,988	National Schedule of NHS Costs—Year 2018–19—NHS trusts and NHS foundation trusts, Elective inpatient for urinary incontinence or other urinary problems, average cost; https://www.england.nhs.uk/national-cost-collection/#ncc1819
Hospital outpatient	39 (0.197)	39 (0.194)	£135	PSSRU 2019, Weighted average of all outpatient attendances; https://www.pssru.ac.uk/project-pages/unit-costs/unit-costs-2019/
GP at surgery	19 (0.096)	20 (0.099)	£39	PSSRU 2019, per surgery consultation lasting 9.22mins including direct care staff costs
GP at home	1 (0.005)	2 (0.01)		PSSRU 2019, used same as per surgery consultation lasting 9.22mins including direct care staff costs but multiplied by 3 so approx. 30 mins appointment
Practice nurse at surgery	5 (0.025)	4 (0.020)	£11	PSSRU 2019, GP Practice Nurse, £42 per hour, no indication of home visit cost or times. 45 mins used as an estimate.
Practice nurse at home	3 (0.015)	0	£32	PSSRU 2019, GP Practice Nurse, £42 per hour, estimated at 15 minutes per appointment
District nurse at home	0	1 (0.005)	£35	PSSRU 2019, Band 6 community nurse, salary £33,411, £46 per hour. Estimated at 45 min per appointment to include travel
Physiotherapist	4 (0.02)	21 (0.104)	£54	PSSRU 2019, one-to-one in community
Occupational therapist	16 (0.08)	3 (0.015)	£78	PSSRU 2019, one-to-one in community
Absorbent pads	8,022 (40)	8,274 (41)	£0.45	Average cost for one pad. Cost calculated over 6 and 12 weeks.

The cost of the intervention, including equipment and staff costs, was calculated as £148.

The average cost for each group (over 12 weeks) for the placebo and intervention groups, calculated as £216 and £212, respectively. These total costs included NHS appointments, pad usage, medication and trial related costs.

The differences in scores from the primary outcome measures and average resource use (NHS and trial costs) were combined to compare the costs and benefits between the placebo and intervention groups. NHS resource use was similar in both groups. Where there was improvement in the primary outcome and an additional cost, the added cost per one-point reduction in urine leakage (ICIQ-UI SF) and in LUT dysfunction (IPSS) was calculated (Table 4).

**Table 4:** Comparison of costs and benefits of TTNS for urine leakage (ICIQ-UI SF) and for lower urinary tract dysfunction (IPSS)

ICIQ-UI SF	12 week average	Average costs	Comparison
Placebo *N* = 200	8.49 (SD = 5.13)	£215.78	Improvement in urine leakage and lower cost for intervention group
Intervention *N* = 199	8.09 (SD = 5.01)	£212.08	
Difference	0.40	£3.70	
I-PSS	12 week average	Average costs	Comparison
Placebo *N* = 190	12.14 SD = 6.19	£215.78	Improvement in lower urinary tract dysfunction and lower cost for intervention group
Intervention *N* = 191	11.27 SD = 5.65	£212.08	
Difference	0.87	£3.70	

Over the 12 weeks, the intervention group showed an improvement in the primary outcome measures with lower resource use.

In reality, it is unlikely that those in the control group would receive any intervention hence a sensitivity analysis was run, which excluded the placebo intervention cost but included the NHS resource use for the placebo group. This resulted in an additional cost for the intervention. Full results are shown in Appendix 3, Supplementary data are available in *Age and Ageing* online.

## Discussion/Conclusion

In this double-blind RCT a significantly lower (better) total IPSS score was found at 6 weeks in the intervention group compared with the control group, although the difference fell short of the threshold for clinically meaningful improvement, defined as 3 points. There was no significant difference between groups in any other outcome measures at any time-point. The subgroup analyses did demonstrate a trend towards greater benefit in men over 65 and this was also cost-effective with lower cost and improved health for the intervention group, but this was not statistically significant.

TTNS treatment was shown to be safe and associated with high compliance and willingness from patients to continue the treatment beyond the trial period.

Unlike motor disorders, bladder dysfunction is non-responsive to levodopa, a precursor of central dopamine, suggesting this dysfunction occurs through complex pathophysiology [28]. Despite its impact on the quality of life of PwP we believe that this is the first double-blind, RCT relating to bladder management [29].

TTNS is a form of peripheral neuromodulation targeted towards symptom relief of OAB and urge urinary incontinence. As well as the possible modulation of the neuronal signals from the spinal cord to the PMC neuromodulation may also have supra-spinal effects, which have been investigated in human studies by studying somatosensory evoked potentials before and after percutaneous tibial nerve stimulation (PTNS) and sacral neuromodulation. The results showed alterations in somatosensory evoked potential [15, 30]. This means that stimulation at the tibial nerve results in modifications of synaptic efficiency through the somatosensory pathway, providing information on the function of somatosensory cortical structures at the spino-thalamic level. Whether such stimulation can influence the effect PD has on bladder control is unknown.

Potentially PTNS may be a more accurate and more intense type of stimulation, yet studies have demonstrated equal effectiveness; a study with 68 participants with overactive detrusor persisting after first- or second-line treatments in a non-superiority trial reported no difference in efficacy between PTSN and TTNS [31]. The protocol of the intervention we used was selected from previous studies, the frequency of twice a week was selected following feedback from PwP in what was acceptable to them.

To minimise the risk of performance bias the intervention and training were standardised as far as possible. Identical equipment was used in a similar pattern over the 6-week intervention period for both groups with the patient or carer being unaware of group allocation. The outcome measures were entered by trial ID number only. The only differences between groups were the electrode position on the ankle (medial in the TTNS group, lateral in the sham group) and intensity of stimulation (highest comfortable intensity > 10 mA in the TTNS group; 2 mA in the sham group). About 72% of the sham group and 51% of the intervention group guessed their group allocation correctly at the end of the intervention period. However, both groups had similar levels of adherence as recorded on the hidden treatment timer, and both reported similar levels of wanting to continue with the use of the device.

No questionnaires have been specifically validated in PD patients and bladder dysfunction. A review published since the trial inception concluded that the IPSS may not be sensitive to change in PwP [32]. The change in score of the IPSS at 6 weeks was 2.4 points but once the results are adjusted to take account of the covariates it was further reduced (1.49). Our sample size justification was based on a study by Ruffion *et al*. [23] with 150 patients with LUTS associated with benign prostatic hyperplasia, who established the mean absolute minimal important difference of 3 points [23]. Although this was a different population a paper by Ragab and Mohammed [33] identified that the total IPSS symptoms score significantly correlated with quality of life in PwP. The authors stated that ‘It is well known that the IPSS questionnaire is not specific for evaluating prostatism in men. This also implies that the IPSS is useful in evaluating voiding dysfunction in neurodegenerative diseases in both men and women’ [34–36]. However further research is required to validate such an outcome measure in PwP. Pavy-Le Traon (32) continue to say that the IPSS does not measure some key problems encountered in PD e.g. incontinence.

A limitation of the study was that some characteristic data regarding the PD patients was not collected, which makes it more difficult to generalise and contextualise findings.

## Message

In summary, TTNS was demonstrated to be safe but, despite a high level of compliance, the evidence of effectiveness for TTNS from this fully powered RCT was limited to a statistically significant difference between groups at 6 weeks, which was not clinically significant [23]. The interest shown by PwP in taking part in our research would clearly indicate a lack of effective interventions. Further fully powered RCTs are required to establish treatments and a clear pathway to help improve the quality of life of PwP by improving their LUTS symptoms.

## Supplementary Material

aa-21-1434-File002_afac114
