# Supplementary material for: Stimulation of the tibial nerve—a randomised trial for urinary problems associated with Parkinson’s—the STARTUP trial
Source: Age Ageing. 2022 Jun 15;51(6):afac114. doi: 10.1093/ageing/afac114 (PMC9200143; doi:10.1093/ageing/afac114)
Supplement: aa-21-1434-File002_afac114 [file aa-21-1434-file002_afac114.docx]

Stimulation of the Tibial Nerve – A randomised trial for urinary problems associated with Parkinson’s - The STARTUP trial

**Supplementary material**

- **Appendix 1:** Quality of life outcomes
- **Appendix 2:** Bladder episodes
- **Appendix 3:** Cost-effectiveness Subgroup Analysis

## Appendix 1: Quality of life outcomes

|  | **Baseline** | | **6 weeks** | | | **12 weeks** | | |
| --- | --- | --- | --- | --- | --- | --- | --- | --- |
|  | **Placebo**  **Arm** | **Intervention Arm** | **Placebo**  **Arm** | **Intervention Arm** | **Effect size** | **Placebo**  **Arm** | **Intervention Arm** | **Effect size** |
|  | *N, Mean (SD)* | *N, Mean (SD)* | *N, Mean (SD)* | *N, Mean (SD)* | *MD (95% CI)* | *N, Mean (SD)* | *N, Mean (SD)* | *MD (95% CI)* |
|  |  |  |  |  |  |  |  |  |
| **PDQ-8 score index** | 106, 30.2 (16.6) | 110, 28.9 (15.9) | 106, 30.7 (15.7) | 100, 28.9 (16.9) | -1.83 (-6.20, 2.54)  p= 0.411 | 94, 29.4 (15.9) | 96, 28.9 (18.4) | -1.00 (-5.79, 3.80)  p=0.684 |
| Mobility | 107, 1.4 (1.3) | 114, 1.3 (1.2) | 108, 1.5 (1.3) | 103, 1.1 (1.2) |  | 96, 1.5 (1.2) | 99, 1.2 (1.2) |  |
| Activities of daily living | 107, 1.4 (1.3) | 114, 1.3 (1.3) | 107, 1.3 (1.2) | 103, 1.3 (1.2) |  | 96, 1.3 (1.1) | 99, 1.2 (1.2) |  |
| Emotional wellbeing | 107, 1.2 (1.0) | 115, 1.0 (1.0) | 108, 1.2 (0.9) | 103, 1.0 (0.9) |  | 96, 1.1 (0.9) | 99, 1.0 (0.9) |  |
| Social support | 106, 0.7 (0.9) | 112, 0.7 (1.0) | 107, 0.9 (1.0) | 100, 0.8 (1.0) |  | 94, 0.8 (0.9) | 96, 0.8 (1.0) |  |
| Cognitions | 107, 1.3 (1.0) | 115, 1.3 (1.1) | 108, 1.3 (1.0) | 103, 1.2 (1.0) |  | 96, 1.3 (1.0) | 99, 1.3 (1.1) |  |
| Communication | 107, 1.1 (1.1) | 114, 1.1 (1.0) | 108, 1.2 (1.0) | 103, 1.2 (1.0) |  | 96, 1.1 (0.9) | 99, 1.3 (1.1) |  |
| Bodily discomfort | 108, 1.5 (1.2) | 114, 1.6 (1.1) | 108, 1.5 (1.1) | 103, 1.4 (1.0) |  | 96, 1.3 (1.1) | 99, 1.5 (1.1) |  |
| Stigma | 108, 1.1 (1.2) | 114, 1.1 (1.1) | 108, 1.0 (0.9) | 103, 1.1 (1.0) |  | 96, 1.1 (0.9) | 99, 1.0 (1.1) |  |
| **SF-Qualiveen overall score** | 103, 1.8 (1.0) | 108, 1.7 (0.9) | 105, 1.7 (1.0) | 101, 1.7 (0.8) | -0.12 (-0.35, 0.11)  p=0.294 | 90, 1.8 (1.0) | 95, 1.7 (0.9) | -0.07 (-0.30, 0.15)  p=0.514 |
| Bother with limitations | 104, 1.4 (1.0) | 112, 1.4 (0.9) | 107, 1.4 (1.0) | 102, 1.4 (1.0) |  | 94, 1.4 (1.0) | 99, 1.4 (1.0) |  |
| Fears | 105, 2.0 (1.2) | 110, 1.8 (1.1) | 106, 2.0 (1.2) | 103, 1.8 (1.0) |  | 95, 1.9 (1.1) | 97, 1.8 (1.1) |  |
| Feeling | 106, 1.8 (1.2) | 112, 1.5 (1.1) | 107, 1.7 (1.2) | 102, 1.5 (1.0) |  | 94, 1.7 (1.2) | 97, 1.6 (1.1) |  |
| Frequency of limitations | 106, 2.0 (1.1) | 112, 2.0 (1.0) | 107, 1.9 (1.1) | 103, 1.9 (1.0) |  | 95, 1.9 (1.1) | 99, 1.9 (1.0) |  |

**Appendix 2: Bladder episodes**

|  | **Baseline** | | **6 weeks** | | | **12 weeks** | | |
| --- | --- | --- | --- | --- | --- | --- | --- | --- |
|  | **Placebo**  **Arm** | **Intervention Arm** | **Placebo**  **Arm** | **Intervention Arm** | **Effect size** | **Placebo**  **Arm** | **Intervention Arm** | **Effect size** |
|  | **N=111** | **N=114** | **N=108** | **N=105** | *MD (95% CI)* | **N=90** | **N=95** | *MD (95% CI)* |
| Micturition episodes (daily) | 9.9 (2.9) | 9.9 (2.4) | 9.3 (2.6) | 9.4 (2.6) | 0.12 (-0.41,0.64) | 9.6 (2.8) | 9.4 (2.6) | -0.21 (-0.79, 0.38) |
| Urgency episodes (daily) | 5.1 (3.8) | 4.5 (3.4) | 3.8 (3.5) | 3.3 (3.4) | -0.23 (-0.93,0.47) | 3.9 (3.8) | 3.4 (3.4) | -0.27 (-0.96, 0.42) |
| Leakage episodes (daily) | 2.4 (3.0) | 2.2 (2.5) | 2.1 (2.9) | 1.8 (2.7) | -0.12 (-0.62,0.37) | 2.0 (2.7) | 2.0 (2.6) | -0.02 (-0.51, 0.47) |
| Leakage severity^1^ | **N=111** | **N=113** | **N=108** | **N=100** |  | **N=88** | **N=94** |  |
| Slight *n, %* | 50 45.0% | 56 49.6% | 42 38.9% | 30 30.0% |  | 32 36.4% | 31 33.0% |  |
| Wet *n, %* | 21 18.9% | 22 19.5% | 20 18.5% | 23 23.0% |  | 18 20.5% | 24 25.5% |  |
| Very wet  *n, %* | 12 10.8% | 10 8.8% | 13 12.0% | 11 11.0% |  | 10 11.4% | 9 9.6% |  |
| Nocturia^1^  *n, %* | 103 92.8% | 106 93.8% | 100 92.6% | 92 92.0% |  | 79 89.8% | 93 98.9% |  |

^1^ Figures for Leakage Severity and Nocturia are shown as a percentage of the number of participants who completed bladder diaries for all 3 days.

Table 3: Comparing the costs and benefits of TTNS for urine leakage (ICIQ-UI SF) with zero cost for trial equipment and staff for the placebo group

| ICIQ-UI SF | 12 week average | Average costs | Comparison |
| --- | --- | --- | --- |
| Placebo  N= 193 | 8.49 | £68.24 | Improvement in urine leakage and additional cost for intervention group |
| Intervention N=195 | 8.09 | £212.08 |  |
| Difference | 0.40 | £143.84 | £359.60 |

Table 4: Comparing the costs and benefits of TTNS for lower urinary tract dysfunction (IPSS) with zero cost for trial equipment and staff for the placebo group

| IPSS | 12 week average | Average costs | Comparison |
| --- | --- | --- | --- |
| Placebo  N=184 | 12.23 | £58.94 | Improvement in lower urinary tract dysfunction and additional cost for intervention group |
| Intervention N=187 | 11.26 | £133.53 |  |
| Difference | 0.97 | £143.84 | £148.29 |

**Appendix 3: Cost-effectiveness Subgroup Analysis**

The cost-effectiveness analysis was repeated using the same subgroups outlined for the main statistical analysis above using the data collected at baseline.

For each subgroup, the average ICIQ-UI SF and IPSS score was calculated and combined with the average resource use from the trial, including the cost of the intervention, over 12 weeks.

**ICIQ-UI SF**

For those with Antimuscarinic medication status of failed, baseline IPSS category of mild and severe, there is an improvement in urine leakage and an additional cost for the intervention group.

Improvement in urine leakage in the intervention group was found for those 65 years plus, males, Antimuscarinic medication status of continuing and failed and baseline IPSS category of moderate, with a lower cost for those groups.

**IPSS**

For females, those with a baseline IPSS category of mild and severe, there is an improvement in lower urinary tract dysfunction and an additional cost for the intervention group

Improvement in lower urinary tract dysfunction in the intervention group was found for those 65 years plus, males, Antimuscarinic medication status of naïve and continuing and baseline IPSS category of moderate, with a lower cost for those groups.
